# Cortical laminar tau deposits and activated astrocytes in Alzheimer’s disease visualised by ^3^H-THK5117 and ^3^H-deprenyl autoradiography

**DOI:** 10.1038/srep45496

**Published:** 2017-04-04

**Authors:** Laetitia Lemoine, Laure Saint-Aubert, Inger Nennesmo, Per-Göran Gillberg, Agneta Nordberg

**Affiliations:** 1 Department of Neurobiology, Care Sciences and Society, Center for Alzheimer Research, Division of Translational Alzheimer Neurobiology, Karolinska Institutet, Stockholm S-14157, Sweden; 2 Department of Pathology, Karolinska University Hospital, Stockholm, Sweden; 3 Department of Geriatrics, Karolinska University Hospital, Huddinge Stockholm, Sweden

## Abstract

Hyperphosphorylated tau protein deposits and, inflammatory processes are characteristic components of Alzheimer disease (AD) pathology. We here aimed to visualize *in vitro* the distribution of tau deposits and activated astrocytes across the cortical layers in autopsy AD brain tissue using the radiotracers ^3^H-THK5117 and ^3^H-deprenyl. ^3^H-THK5117 and ^3^H-deprenyl autoradiographies were carried out on frozen brain sections from three AD patients and one healthy control. ^3^H-THK5117 showed a distinct laminar cortical binding similar to ^3^H-deprenyl autoradiography, with an extensive binding in the superficial and deep layers of the temporal neocortices, whereas the middle frontal gyrus showed an even binding throughout the layers. Globally, eventhough some differences could be observed, AT8 (tau) and GFAP (astrocyte) immunostaining showed a laminar pattern comparable to their corresponding radiotracers within each AD case. Some variability was observed between the AD cases reflecting differences in disease phenotype. The similar laminar cortical brain distribution of tau deposits and activated astrocytes supports the hypothesis of a close pathological interconnection. The difference in regional binding patterns of ^3^H-THK5117 and AT8 antibody staining suggest additional tau binding sites detectable by ^3^H-THK5117.

Alzheimer disease (AD) is characterised by several pathological processes such as accumulation of misfolded proteins as well as neuroinflamation processes. The patterns of progression for amyloid-beta (Aβ) plaques and hyperphosphorylated tau deposits (neurofibrillary tangles – NFTs) in AD, assessed according to specific stereotypical stages, appear to be different. While Aβ deposition starts early, first affecting the neocortex and then spreading to the allocortex and subcortical structures^1^, NFTs first occur in the transentorhinal and entorhinal regions of the medial temporal lobe, then extend outwards to other limbic regions, and finally reach the neocortical regions^2^. However, while the spreading pattern of tau pathology in AD is well characterised, its interactions with other pathological hallmarks of the disease are not yet fully understood.

Neuroinflammation processes affect non-neuronal glial cells located in both white and grey matter during the course of AD. Activated astrocytes, which are known to play a major role in brain homeostasis, have different activation states according to their location relative to Aβ plaques. The astrocytes close to Aβ plaques are activated and go through gliosis while those further from the plaques are in an apoptotic state^3^,^4^. In their recent review, Pekny and colleagues^5^ described different aspects of activated astrocytes and offered a hypothesis of their role in neurological diseases. Activated astrocytes are thought to possess both a neurodegenerative and a ‘protective’ role in AD^5^. In a recent positron emission tomography (PET) study, *in vivo* astrocytosis (measured with the monoamine oxidase B (MAO-B) inhibitor ^11^C-L-deprenyl and amyloid accumulation (measured with ^11^C-Pittsburgh compound B (^11^C-PIB) had divergent time courses, with the highest ^11^C-deprenyl binding occurring 20 years before the expected onset of clinical symptoms^6^. The relative timing of tau deposition and astrocytosis in AD is still under investigation. The numbers of both activated astrocytes and microglia correlate with the numbers of NFTs at different stages of the disease, with the association increasing in the later stages^7^. However, activation of the astrocytes does not seem to be sufficient to reduce the burden of the tau deposits^8^.

The recent development of specific ligands for tau offers new tools for studying the underlying mechanisms of tau pathology. Recently, several tau PET tracers with different chemical structures have been developed (for review see refs 9 and 10); these include THK derivatives. In a previous study from our group, saturations binding studies have demonstrated affinity of ^3^H-THK5117 in the nM range in AD brain tissue, and competition binding studies have revealed at least three binding sites, in the pM, nM and μM ranges respectively^11^. In the same study, we showed good regional correspondence between ^3^H-THK5117 binding using autoradiography and AT8 tau immunostaining in brain tissue from three AD cases^11^. From this study, we could already observe visually a heterogeneous – laminar – binding throughout the cortex of those cases. This binding seemed to reflect the laminar distribution of NFTs, that primarily affect pre-α and pri-α layers in the entorhinal cortex^12^, and layers III and V in the neocortex^13^,^14^,^15^. Importantly, during the time course of AD NFTs evolve from intracellular pretangles to extracellular ghost tangles^16^, and affect connected layers in a sequential manner, indicating that neuronal connectivity has a critical role in the propagation of tau^17^. *In vivo* PET studies have shown that (*S*)-^18^F-THK5117 shows significantly higher retention in cortical and subcortical brain regions of AD patients in comparison to controls^18^. Since PET imaging has a low spatial resolution and only reflects the total binding of a ligand, it does not allow detailed comparisons with histopathology. It is therefore important to address the question of the correspondence between tau PET ligands in frozen brain tissue and a validated antibody to relate what is observed *in vivo* and in histopathology, and to appreciate to which type(s) of NFTs it binds to.

In the present pilot study, we aimed to quantify and compare, in the three AD cases previously described^11^, the cortical laminar distribution of tau deposits and activated astrocytes using ^3^H-THK5117 and ^3^H-deprenyl autoradiographies on frozen hemispheres. In addition, we also aimed to compare their cortical laminar distributions with the corresponding regional AT8 and Glial Fibrillary Acidic Protein (GFAP) immunostaining.

## Results

### Visual assessment of cortical laminar distribution using autoradiography and immunostaining

**Figure 1:**
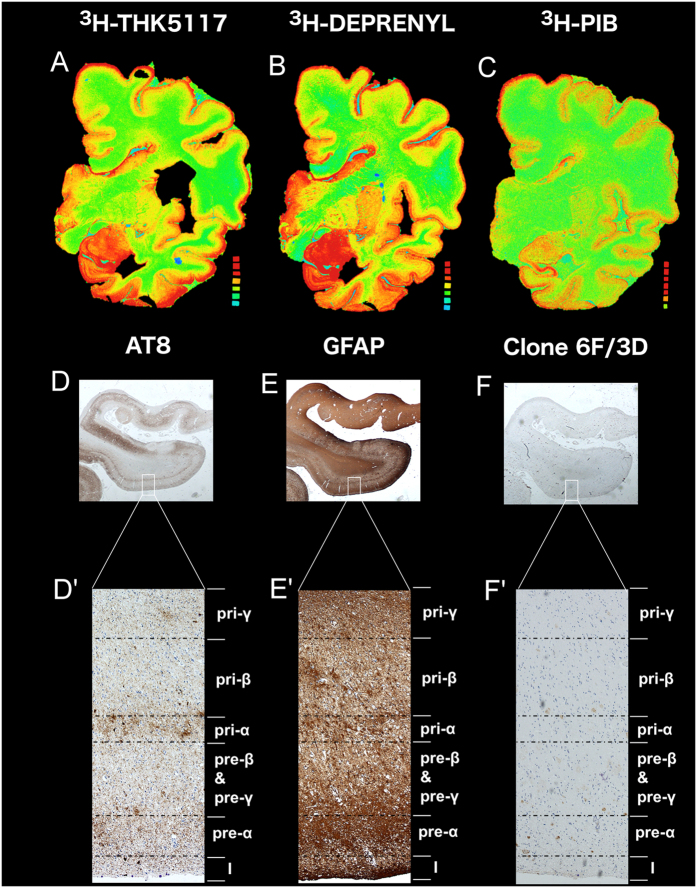
Study layout illustrated in AD case 1. Autoradiography with ^3^H-THK5117 (**A**), ^3^H-deprenyl (**B**) and ^3^H-PiB (**C**) on frozen large hemisphere samples from AD case 1. Immunostaining on paraffin sections from the corresponding contralateral hemisphere in the entorhinal gyrus with AT8 (**D**), GFAP (**E**) and Clone 6F/3D (**F**). A zooming on the different layers is presented in D’, E’ and F’ for AT8, GFAP and clone 6F/3D, respectively.

Visual assessment of the large frozen-section autoradiograms showed a laminar pattern of ^3^H-THK5117 and ^3^H-deprenyl binding, especially in the temporal regions, while no laminar pattern was observed for ^3^H-PiB. The corresponding immunostaining on paraffin sections from the right hemisphere showed a similar pattern. As an example, Fig. 1 shows the autoradiograms as well as the medial temporal immunostainings for AD case 1.

**Figure 2:**
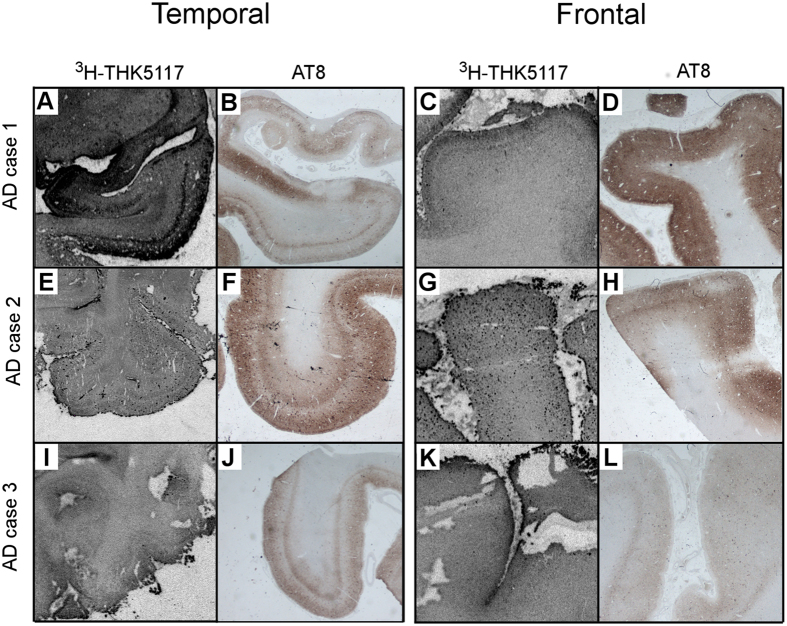
Correspondence between ^3^H-THK5117 autoradiography and AT8 staining. Autoradiography on frozen sections from AD brains 1, 2 and 3 using ^3^H-THK5117 are respectively shown for the temporal cortex in (**A**,**E**,**I**) and for the frontal cortex in (**C**,**G**,**K**). Corresponding AT8 immunostaining on paraffin section from the contralateral hemisphere is presented in (**B**,**F**,**J** and **D**,**H**,**L**) for temporal and frontal cortex, respectively.

In the temporal gyrus of all three AD cases, ^3^H-THK5117 autoradiography (Fig. 2A,E and I) and AT8 staining (Fig. 2B,F and J) showed similar laminar distribution, with binding in superficial and deep layers of the cortex. Similar findings were observed in the frontal cortex but only in case 1 (Fig. 2C,D).

On visual assessment of the ^3^H-THK5117 and ^3^H-deprenyl autoradiograms, a superficial and a deep band of binding were observed in the temporal ROIs (Supplementary Fig. S1). This pattern was most prominent in the entorhinal cortex of AD case 1 (Supplementary Fig. S1A, SA’) but was also seen in the fusiform gyrus of AD cases 2 and 3 (Supplementary Fig. S1B,B’,C,C’). Only the superficial binding band was observed in the frontal region (Supplementary Fig. S2), except for AD case 1, where a faint deep layer band was observed (Supplementary Fig. S2A,A’). No laminar pattern was observed for the two ligands in the control case (Supplementary Fig. S1D,D’ and Supplementary Fig. S2D,D’).

### Quantitative assessment of cortical laminar distribution on large frozen brain hemisphere autoradiograms

For each case, different selections have been created in each of the four ROIs and are shown in yellow in Supplementary Figs S1 and 2.

**Figure 3:**
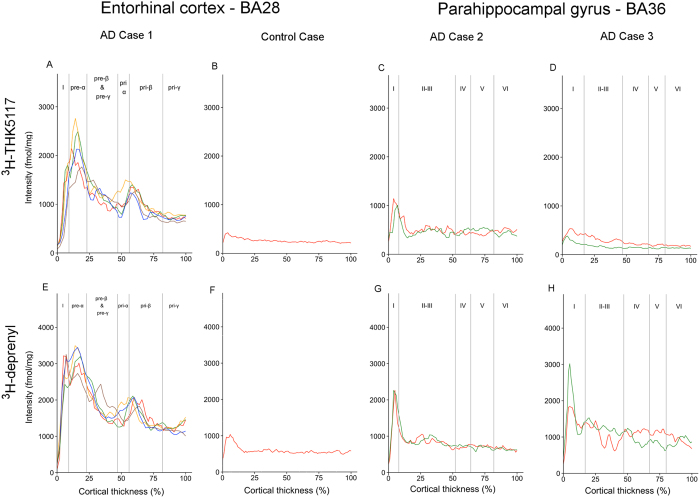
Laminar binding profiles of ^3^H-THK5117 and ^3^H-Deprenyl from selections within the entorhinal cortex (BA28) and parahippocampal gyrus (BA36). The ^3^H-THK5117 binding profiles are presented in (**A**,**B**,**C**,**D**) (**A**) AD case 1, (**B**) control case, (**C**) AD case 2, and (**D**) AD case 3. ^3^H-Deprenyl binding profiles are presented in (**E**,**F**,**G**,**H**) (**E**) AD case 1, (**F**) control case, (**G**) AD case 2 and (**H**) AD case 3. The different colors correspond to different selection in the region of interest.

**Figure 4:**
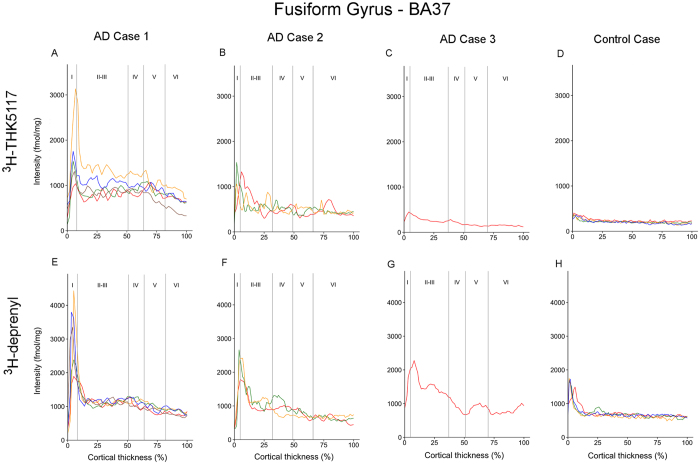
Laminar binding profiles from selections within the fusiform gyrus (BA37). ^3^H-THK5117 binding profiles are presented in (**A**,**B**,**C**,**D**) (**A**) AD case 1; (**B**) AD case 2; (**C**) AD case 3 and (**D**) control case 4. ^3^H-Deprenyl binding profiles are presented in (**E**,**F**,**G**,**H**) (**E**) AD case 1; (**F**) AD case 2; (**G**) AD case 3 and (**H**) control case. The different colors correspond to different selection in the region of interest.

**Figure 5:**
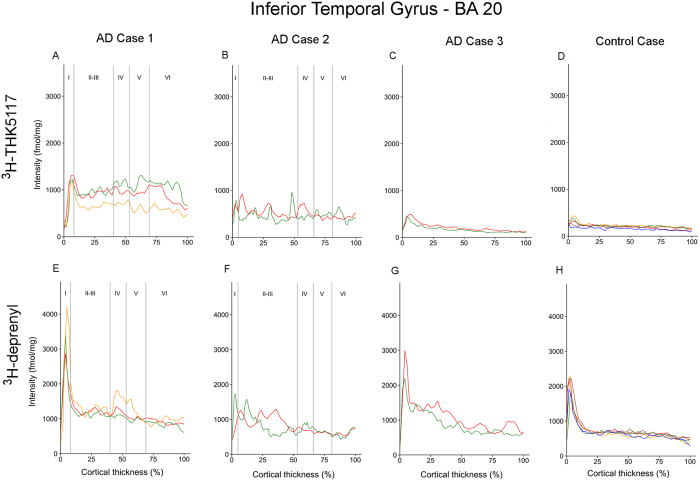
Laminar binding profiles from selections within the inferior temporal gyrus (BA20). ^3^H-THK5117 binding profiles are presented in (**A**,**B**,**C**,**D**) (**A**) AD case 1; (**B**) AD case 2; (**C**) AD case 3 and (**D**) control case. ^3^H-Deprenyl binding profiles are presented in (**E**,**F**,**G**,**H**) (**E**) AD case 1; (**F**) AD case 2; (**G**) AD case 3 and (**H**) control case. The different colors correspond to different selection in the region of interest.

**Figure 6:**
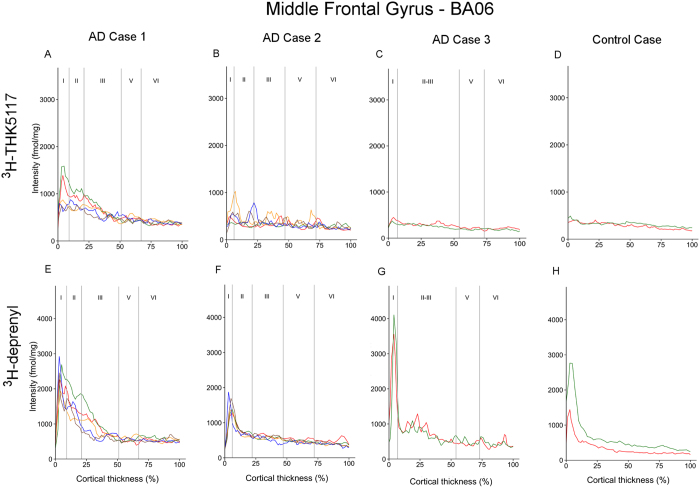
Laminar binding profiles from selections within the middle frontal gyrus (BA06). ^3^H-THK5117 binding profiles are presented in (**A**,**B**,**C**,**D**) (**A**) AD case 1; (**B**) AD case 2; (**C**) AD case 3 and (**D**) control case. ^3^H-Deprenyl binding profiles are presented in (**E**,**F**,**G**,**H**) (**E**) AD case 1; (**F**) AD case 2; (**G**) AD case 3 and (**H**) control case. The different colors correspond to different selection in the region of interest.

The regional laminar binding profiles of ^3^H-THK5117 and ^3^H-deprenyl in AD cases 1, 2, 3 and control case are presented in Figs 3–6. The total binding was used in the analyses since there were no differences in binding pattern between specific and total binding methods (Supplementary Fig. S3). There was generally good consistency between the profiles of the different selections within each ROI.

Some general observations can be made. The binding of ^3^H-deprenyl was more extensive than that of ^3^H-THK5117. No laminar binding pattern was observed in the five ROIs for the control case except for a narrow peak for ^3^H-deprenyl binding in a very superficial layer. This superficial peak was highest in the inferior temporal gyrus, with a density around 2000 fmol/mg (Fig. 5H), and the middle frontal gyrus, with a density between 1500–2800 fmol/mg (Fig. 6H).

In order to assess the selectivity of the tracers towards each other, additional autoradiography competitions experiments were performed between ^3^H-THK5117 and unlabeled deprenyl as well as between ^3^H-deprenyl and unlabeled THK5117. No competition was observed in the nanomolar range concentration (results are presented in Supplementary Data 1).

#### Entorhinal/parahippocampal gyrus

**Figure 7:**
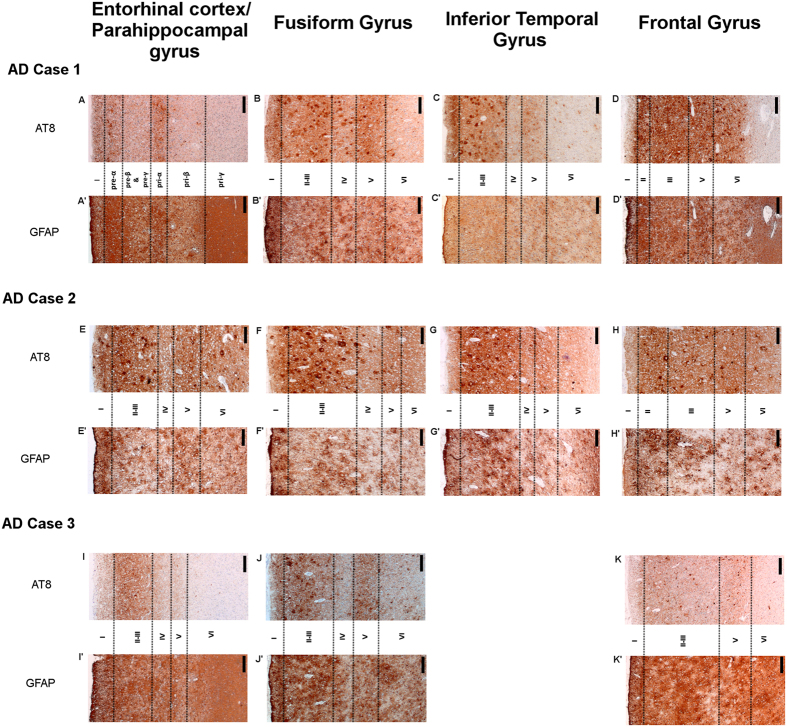
Correspondence between AT8 and GFAP staining in paraffin sections. Immunostaining in each regions of interest is shown for the three AD cases. For AD Case 1, AT8: **A–D** and GFAP: **A’–D’**. For AD Case 2, AT8: **E–H** and GFAP: **E’–H’**. For AD Case 3, AT8: **I–K** and GFAP: **I’–K’**. Note that staining in the inferior temporal was not available for case 3. ×40 zoomed, scale bar = 100 µm.

In the entorhinal cortex of AD case 1, there was a wide superficial peak in ^3^H-THK5117 binding located in the pre-α layer (binding density 1765–2759 fmol/mg) and a second peak in the deeper pri-α/pri-β layers (binding density around 1200–1400 fmol/mg; Fig. 3A). A similar binding pattern was observed for ^3^H-deprenyl binding, with higher intensity in both peaks (binding density 3193–3495 fmol/mg and around 2000 fmol/mg for the superficial and deep peaks, respectively; Fig. 3E). The observed peaks roughly matched the corresponding AT8 and GFAP staining bands from paraffin sections (Fig. 7A,A’). In the parahippocampal region of AD case 2, the binding patterns of ^3^H-THK5117 and ^3^H-deprenyl were similar, with narrow peaks in the superficial layer I (Fig. 3C,G). Although the binding intensity in the peak was twice as high for ^3^H-deprenyl, the intensity through the deep cortical layer was similar for both ligands (binding density 500 fmol/mg). AT8 staining showed rounder, larger tau deposits in AD case 2 (Fig. 7E,E’) than in AD case 3 (Fig. 7E,I). The binding patterns for ^3^H-THK5117 and ^3^H-deprenyl in the parahippocampal gyrus of AD case 3 were different. The binding density of the superficial narrow peak in layer I for ^3^H-deprenyl was 1838–3020 fmol/mg (Fig. 3H) while the maximum binding density for ^3^H-THK5117 was 533 fmol/mg (Fig. 3D). No clear laminar pattern was observed for GFAP staining, while there was a large band of AT8 staining, starting from layer II (Fig. 7I,I’).

#### Fusiform gyrus

In the fusiform gyrus of AD case 1, the ^3^H-THK5117 and ^3^H-deprenyl autoradiograms showed high binding of different intensities for the first peak in layer I (binding density 1037–3134 fmol/mg and 1893–4433 fmol/mg, respectively; Fig. 4A,E). For both ^3^H-THK5117 and ^3^H-deprenyl, this first peak was very narrow and there was no second peak, but the binding density remained high throughout the deeper layers, at around 1000 fmol/mg. This high binding intensity matched the immunostaining pattern for AT8 in the superficial layer, while almost no staining was observed in the deeper layers (Fig. 7B). GFAP staining was observed on the border of the cortex, and more homogeneous binding was seen through the cortical layers (Fig. 7B’). The fusiform gyrus of AD case 2 showed extensive binding of ^3^H-THK5117 in superficial layers I-II (Fig. 4B) with a binding density around 1500 fmol/mg, which dropped to 500 fmol/mg in the deeper cortex. A similar binding pattern was observed with ^3^H-deprenyl, with a higher intensity for the superficial peaks in layer I through the entire cortex (Fig. 4F). The AT8 immunostaining results for the fusiform gyrus (Fig. 7F) showed a patchy pattern with round tau deposits. GFAP staining reflected the plot observed with ^3^H-deprenyl well (Fig. 7F’). The binding profiles were different for ^3^H-THK5117 and ^3^H-deprenyl in the fusiform gyrus of AD case 3, although it should be taken into account that only one selection was possible in this ROI. AD case 3 showed low levels of ^3^H-THK5117 binding (Fig. 4C); the maximum binding density of the first peak in the superficial layer was 492 fmol/mg and that of the second peak was close to 100 fmol/mg. The corresponding AT8 immunostaining in the fusiform gyrus (Fig. 7J) showed a clear binding pattern in superficial and deep layers that was not observed in the ^3^H-THK5117 plot. A superficial peak was observed in the external part of layer II on the ^3^H-deprenyl binding plot (Fig. 4G); the binding density was 2186–2990 fmol/mg in layers II-III, dropping to 1000 fmol/mg in the deepest layer.

#### Inferior temporal gyrus

In the inferior temporal gyrus of AD case 1 (Fig. 5A,E), the first high peak in layer I had different binding intensities for the ligands (binding density 1192–1311 fmol/mg for ^3^H-THK5117 and 2875–4208 fmol/mg for ^3^H-deprenyl). There was a small decrease in ^3^H-THK5117 binding intensity in layers II-III, but it increased again in deeper layers (binding density around 1000 fmol/mg). No clear peaks were identified for ^3^H-THK5117 and ^3^H-deprenyl in layers IV-V. The ^3^H-THK5117 binding pattern seemed to be similar to that seen with AT8 immunostaining, with high binding in layer I, some tau deposits in layers II-III, almost no binding in layer IV and more tau deposits in layer V (Fig. 7C). The GFAP staining was more homogeneous through the cortical layers (Fig. 7C’). In the inferior temporal gyrus of AD case 2, the profiles for ^3^H-THK5117 (Fig. 5B) and ^3^H-deprenyl (Fig. 5F) were more patchy, with binding densities oscillating between 790 and 1724 fmol/mg, without clear peaks. AT8 immunostaining showed a patchy pattern with round tau deposits (Fig. 7G), while GFAP staining reflected the plot observed for ^3^H-deprenyl well (Fig. 7G’). The binding profiles for ^3^H-THK5117 and ^3^H-deprenyl in the inferior temporal gyrus of AD case 3 showed similar patterns to those in the fusiform gyrus (Fig. 5C,G).

#### Middle frontal gyrus

In the middle frontal gyrus, the maximum binding density was 1586 fmol/mg for AD case 1 and <1000 fmol/mg for AD cases B and C and the control case. ^3^H-THK5117 binding in the deep layers was homogeneous, with no laminar patterns in any of the AD brains (Fig. 6A–C), comparable to the control case (Fig. 6D). The binding of ^3^H-deprenyl of AD case 1 and B was similar to that of ^3^H-THK5117 in all the layers, with the first high intensity peak in superficial layer I (binding density 1900–3000 fmol/mg). There was uniform binding in the deeper layers, with lower intensity (binding density around 500 fmol/mg) in all cases (Fig. 6E–H). AD case 1 had wide superficial ^3^H-THK5117 (Fig. 6A) and ^3^H-deprenyl (Fig. 6E) peaks through layers I to III, while cases B and C had no superficial peak for ^3^H-THK5117 and only a narrow peak in layer I for ^3^H-deprenyl (Fig. 6F,G). Both AT8 and GFAP showed a laminar pattern with a band in the superficial and deep layers in AD cases B and C (Fig. 7H,H’), but not AD case 1 (Fig. 7D,D’).

## Discussion

In this pilot study, we investigated the relationships between tau deposits and activated astrocytes in AD by assessing and quantifying their laminar cortical distribution using ^3^H-THK5117 and ^3^H-deprenyl autoradiography. To the best of our knowledge, this study is the first one to assess the layer distribution of a tau PET tracer in frozen brain tissue.

Visual assessment of the *in vitro*^3^H-THK5117 and ^3^H-deprenyl autoradiographs revealed comparable laminar profiles in most ROIs for AD cases 1 and 2, with the exception of inferior temporal gyrus for AD case 1 and middle frontal gyrus for AD case 2. The results for AD case 3 were more heterogeneous across the ROIs. These findings were further supported by similar staining patterns for AT8 and GFAP on matched paraffin sections, indicating an association between tau deposits and activated astrocytes. In agreement with a previous study^19^, while ^3^H-deprenyl binding reached a high peak in the superficial layers, we observed an even distribution of ^3^H-PiB binding through the cortical layers, suggesting a different laminar progression pattern for activated astrocytes and Aβ plaques, which may be related to a different time course for these pathological processes.

The quantitative analyses of ^3^H-THK5117 laminar binding profiles revealed a peak in the superficial layers, of varying densities, in the temporal regions of all three AD cases. For some regions, a second peak was observed in the deeper layers. Of all the ROIs analysed, the second ^3^H-THK5117 peak was most clearly observed in the entorhinal cortex of AD case 1. Using the corresponding AT8 immunostaining samples, we observed that the more superficial laminar peak was located in the pri-α/pri-β layer and the deeper peak was located in the pre-α layer. These results are in agreement with the known distribution of NFTs in the entorhinal cortex of AD brains^20^.

Overall, extensive ^3^H-deprenyl binding was visually observed in the AD cases. Our quantitative analyses revealed high binding peaks in layer I, in agreement with the reported presence of interlaminar astrocytes in this layer^21^, where activation is thought to play an early role in AD pathogenesis and to contribute to Aβ plaque formation^22^. Indeed, *in vivo* PET studies using ^11^C-deprenyl have found prominent astrocytosis from the early clinical stages of AD, suggesting that astrocytosis is an early event in the AD time course^23^,^24^, possibly even preceding the deposition of Aβ plaques^25^,^26^. ^3^H-deprenyl binding was also observed in deeper layers in the temporal ROIs of the AD cases. Interestingly, we also observed superficial ^3^H-deprenyl binding in the control case, in contrast to the absence of ^3^H-THK5117 binding. The terminal ends of astrocytes are known to be located in the glia limitans of the healthy brain, providing a physical barrier between the cortex and the meninges^27^,^28^, which could partly explain the superficial ^3^H-deprenyl binding.

No or minor competition was observed between ^3^H-THK5117 and unlabeled deprenyl as well as between ^3^H-deprenyl and unlabeled THK5117 in the range of concentration we are using for the autoradiography (i.e nanomolar range). This finding is of critical importance as, for the first time, it brings evidence that, at those concentrations, the tau tracer THK5117 does not bind to MAO-B. The close resemblance between our quantified ^3^H-THK5117 and ^3^H-deprenyl laminar profiles would indeed indicate a laminar association between tau deposits and activated astrocytes. Previous *in vitro* studies have demonstrated close localizations between microglia, astroglia and tangle-bearing neurons using immunohistochemistry techniques in AD brain tissue at different stages of the disease. Using IL-1α (microglia), S100β (astrocytes) and tau2 (tangle containing) antibodies, Sheng *et al*. demonstrated a positive correlation between numbers of IL-1α^+^ and S100β^+^ and number of tau2^+^, along with augmentation of the NFTs stages following the same pattern^7^. Moreover, extracellular tau deposits (i.e. ghost tangles) strongly attract astrocytes in AD brains^29^. All these observations could account for the laminar co-localization observed here.

It has been proposed that tau should be classified as a “prion-like” protein, with an ability to spread through neural connections^30^, as demonstrated in transgenic mice between synaptically linked neurons^1^,^8^,^31^. Post-mortem analyses of AD tissue at different stages have described tau deposition in the terminal zones of known cortical projections^17^. Given that cortical layers play a crucial role in the organisation of these projections, the accumulation of tau in the projections of specific cortical layers could be an important facilitating factor in the spread of tau from one region to another. However, the relationship between these layers and brain connectivity is complex and no conclusions about the spreading patterns of tau could be drawn from our data.

The different laminar patterns of NFTs for the five ROIs in this study have been well described in the literature. In 2008, Thangavel *et al*. described the pathology in the fusiform gyrus (BA37)^32^ and the posterior parahippocampal gyrus^33^ in detail. In the fusiform gyrus, the NFTs appear in a peculiar pattern and are distributed in the pyramidal cells of layers III and V. Although all the AD cases in our study were in late Braak tau stages, the laminar profiles for both tau and activated astrocytes were similar within but different across cases. Several factors may have been involved; for example, the more ’patchy’ binding and staining (Figs 2 and 7) may have been responsible in AD case 2. ^3^H-THK5117 binding intensity was much lower in AD case 3 than in AD cases 1 and 2 and no binding peaks were distinguished on the corresponding plots. However, a band in the deep layer was visually observed with AT8 staining, suggesting that, although the distribution patterns were generally similar for ^3^H-THK5117 binding and AT8 staining, these ligands were probably binding different targets. While the AT8 staining site on the tau protein is known^34^, the forms of tau and specific site(s) to which ^3^H-THK5117 is binding remain to be elucidated for the *in vivo* PET application. Preliminary findings however suggest that THK5117 would bind to mature and ghost tangles but not to pre-tangles^35^, while AT8 antibody binds to pretangles but not to late ghost tangles^36^. A recent study using the tau tracer ^18^F-AV1451 also reported that the binding of this PET tracer varied depending on the maturity stage of the tangles^37^. Different antibodies are available to reveal different stages of maturation of the tangles, such as CP13 or PHF-1. To the best of our knowledge, it is not known whether the laminar distributions of the different isoforms or phosphorylation states of tau differ, and it would be of great value to compare ^3^H-THK5117 binding with these tau antibodies in complement to AT8 in the future.

The routine pathology methods used in the present study in AD cases 2 and 3 also demonstrated other pathological features including amyloid angiopathy and Lewy bodies, which may influence tau and/or astrocyte deposition. Recent additional information regarding AD case 1 familial history suggest for a familial form of AD and might account for a different pathological pattern in this case in comparison to AD case 2 and 3.

The present study shows a laminar distribution of ^3^H-THK5117 through the cortex within AD frozen brains reflecting the different distribution of tangles across the cortical layers. We also demonstrated a similar laminar binding pattern for ^3^H-deprenyl tracer through the temporal cortical layers, indicating that tau deposits and inflammatory processes are closely spatially related in AD pathology, possibly reflecting their close pathological interconnection. The differences in laminar patterns observed between the AD cases studied may be explained by their different clinical phenotypes. Our study illustrates the ability of tau and activated astrocytes PET ligands to reveal the laminar distribution of their targets *in vitro*, and suggest that complementary information can be obtained in comparison to immunohistochemistry.

## Materials and Methods

### Chemicals

1-Fluoro-3-((2-(4-([3H]methylamino)phenyl)quinolin-6-yl)oxy)propan-2-ol (3H-THK5117) was custom synthesized by Quotient Bioresearch (Cardiff, UK; specific activity (SA) = 83 Ci/mmol). N-methyl-N-(2-propynyl)-2-methyl-1-phenylethyl-2-amine (3H-L-deprenyl) was custom synthesized by Quotient Bioresearch (Cardiff, UK; SA = 85 Ci/mmol). methyl-3H-Deprenyl (3H-L-deprenyl) will be cited as 3H-deprenyl in the paper. N-methyl-[3H]2-(4’-methylaminophenyl)-6 hydroxybenzothiazole (3H-PiB) was custom synthesized by Novandi (Södertälje, Sweden; SA = 81 Ci/mmol).

### Brain tissue

Brain tissue was obtained from three AD patients (AD case 1: female, 60 years old at death, early onset of the disease, *APOE* ε4/ε4, Braak stages V-VI; AD case 2: female, 79 years old at death, late onset of the disease, *APOE* ε4/ε4, Braak stages V; AD case 3: male, 81 years old at death, late onset of the disease, *APOE* ε4/ε4, Braak stages V-VI). All the AD cases come from the Brain Bank at Karolinska Institutet (Sweden). All three AD cases underwent memory assessment at the memory clinic, Karolinska University Hospital Huddinge, and were clinically longitudinally followed-up. All received cholinesterase inhibitor and memantine treatment. For further details on the cases, see Lemoine *et al*.^11^. In addition, brain tissue from one healthy control (control case age at death: 76 years; Braak tau stage: I) from the Neuropathology of Dementia Laboratory of Indiana University (USA) was used. The left hemisphere of each case was frozen in large block sections while the right hemisphere was fixed in formaldehyde and small blocks were collected and embedded in paraffin for microscopy. For the study, for each AD case the left frozen brain hemisphere was used for autoradiography and the right paraffin embedded hemisphere for immunohistochemistry. A strong positive correlation has been demonstrated between right and left hemisphere for Braak tau staging from a very large set of patients^38^, suggesting a good bilateral correspondence. We are however aware that, to some extent, asymmetry may be observed in tau staining in some cases^39^.

For all three cases, routine pathological assessments using AT8, GFAP and clone 6 F/3D antibodies were performed. The patients and relatives have provided written permission and informed consent for brain autopsie and pathological analysis. All experiments on autopsied human brain tissue are in accordance with ethical permission obtained by the Ethics committee in Stockholm (Permission number 2011/962/31-1).

### Autoradiography on frozen sections from the whole left brain hemisphere

Eighty microns-thick frozen brain sections from the whole left hemisphere were obtained as described previously^40^. The autoradiography experimental procedure was started after allowing the sections to reach room temperature. After 10 minutes of pre-incubation with PBS + 0.1% BSA buffer (pH 7.4), the binding reaction was initiated by incubating the sections with 4 nM ^3^H-THK5117 for 60 minutes. Non-specific binding was determined on an adjacent section by adding 10 μM unlabeled THK5117 to the 4 nM ^3^H-THK5117. A similar protocol was used for ^3^H-deprenyl and ^3^H-PiB sections: ^3^H-deprenyl sections were incubated in Na-K phosphate buffer (pH 7.4) containing 10 nM ^3^H-deprenyl for 60 minutes. Non-specific binding was determined on an adjacent section by adding 1 μM unlabeled deprenyl to the 10 nM ^3^H-deprenyl. ^3^H-PiB sections were pre-incubated for 10 minutes in PBS + 1% BSA buffer (pH 7.4), and then incubated with 1 nM ^3^H-PiB for 45 minutes. Non-specific binding was determined on an adjacent section by adding 10 μM BTA-1 to the 1 nM ^3^H-PiB. All reactions were terminated by washing the sections for 5 minutes in cold buffer three times, and then dipping them once in cold distilled water. The sections were allowed to dry for at least 24 hours and were then exposed on phosphoplates in autoradiography cassettes with tritium standards (Larodan Fine Chemical AB, Malmö, Sweden), for 4 days for ^3^H-THK5117 and ^3^H-deprenyl and for 10 days for ^3^H-PiB. Finally, digitized images of the sections were made with a Fujifilm BAS-2500 scanner.

### Assessment of the tracers’ selectivity

Competition autoradiography studies were performed on small frozen sections in order to check the selectivity of our tracers towards each other. Similar protocol as described previously was applied (see Supplementary Data 1).

### Quantitative assessment of ligands on the large frozen autoradiograms

#### Regions of interest (ROIs)

Cortical ROIs in the four cases were labeled on the digitized autoradiography sections after a consensus of 4 raters with the guidance of an anatomical atlas^31^. Corresponding Brodmann areas (BA) were identified using the classical Brodmann atlas^41^ and a magnetic resonance imaging (MRI)-based atlas^42^. The boundaries between grey and white matter in these ROIs were defined using Luxol fast blue and GFAP-stained paraffin sections (see below).

In order to obtain accurate information on the laminar distribution of THK5117, the study focused on five cortical brain regions, namely the entorhinal gyrus (Brodmann area 28; BA28), parahippocampal gyrus (BA36), fusiform gyrus (BA37), inferior temporal gyrus (BA 20) and middle frontal gyrus (BA06). These regions are known to be involved in different cognitive processes^43^,^44^,^45^,^46^,^47^, and all are impaired at different stages of AD progression, the latter being affected late by tau pathology^2^.

Because of variations in the cutting of the frozen brains, entorhinal cortex was only available for AD case 1 and the control case, while parahippocampal gyrus was only available for AD cases 2 and 3.

#### Distribution analysis

The cortical selections for each case and each ligand were defined in the five ROIs on the digitized autoradiograms of total binding, using ImageJ 1.49q software^48^. Each selection consisted of a 15-pixel wide rectangle, drawn from the cortical surface and extending perpendicularly to the grey and white matter boundary. Up to five selections were defined per ROI but, in some ROIs, the number of selections was limited because of loss of tissue integrity. Each selection was replicated on the corresponding non-specific autoradiogram. For each selection, the density of the grey matter for each 15-pixel row was averaged and then converted into fmol/mg using a standard curve based on tritium standard concentrations. The average binding density across cortical layers, expressed in fmol/mg, was plotted using ggplot2 package and R software. The corresponding boundaries and labels of the cortical layers could not be defined directly on the frozen sections, because of the low resolution of the autoradiograms^49^, and were thus defined from the immunostaining images (see below). It should be noted that this transpositioning of the labels on paraffin sections onto the frozen section images might have led to slight imprecisions.

### Immunostaining on small paraffin sections from the right hemispheres of AD brains

AT8 (tau), GFAP (astrocytes) and Clone 6F/3D (amyloid) immunostaining was performed using routine pathology methodology on adjacent small paraffin sections of AD cases 1, 2 and 3. The distribution of tau deposits, activated astrocytes and Aβ plaques, as measured respectively by AT8, GFAP and Clone 6F/3D staining, was then visually assessed in each neuronal layer.

## Additional Information

**How to cite this article:** Lemoine, L. *et al*. Cortical laminar tau deposits and activated astrocytes in Alzheimer’s disease visualised by ^3^H-THK5117 and ^3^H-deprenyl autoradiography. *Sci. Rep.* **7**, 45496; doi: 10.1038/srep45496 (2017).

**Publisher's note:** Springer Nature remains neutral with regard to jurisdictional claims in published maps and institutional affiliations.

## Supplementary Material

Supplementary Dataset 1
