# Supplementary material for: Cortical laminar tau deposits and activated astrocytes in Alzheimer’s disease visualised by 3H-THK5117 and 3H-deprenyl autoradiography
Source: Sci Rep. 2017 Apr 4;7:45496. doi: 10.1038/srep45496 (PMC5379625; doi:10.1038/srep45496)
Supplement: Supplementary Dataset 1 [file srep45496-s1.doc]

Supplementary Files

**Cortical laminar tau deposits and activated astrocytes in Alzheimer’s disease visualized by ^3^H-THK5117 and ^3^H-deprenyl autoradiography**

Laetitia Lemoine ^1^, Laure Saint-Aubert ^1^, Inger Nennesmo ^2^, Per-Göran Gillberg ^1^, Agneta Nordberg ^1,3^


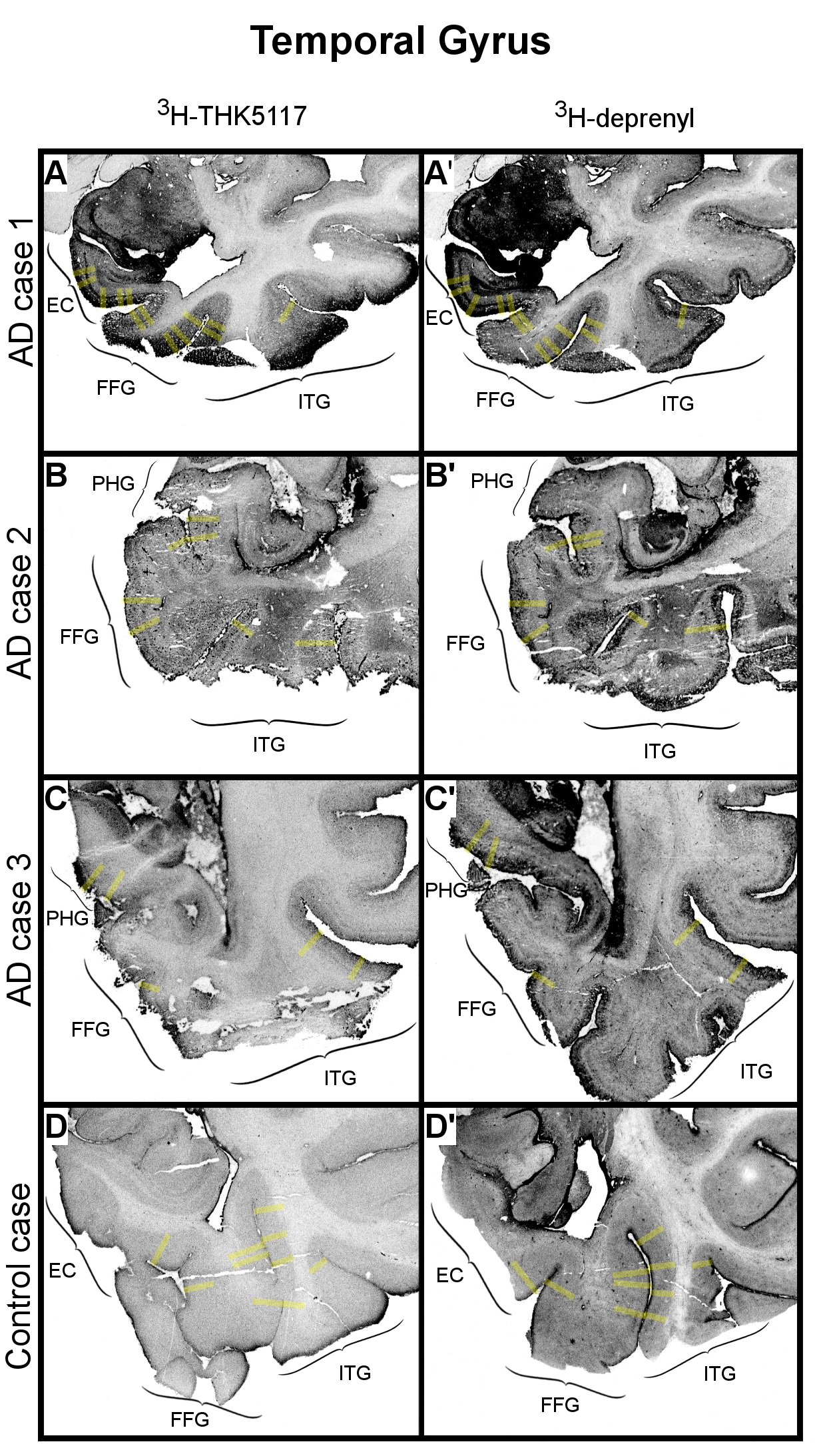


**Supplementary Figure S1.** Autoradiography with ^3^H-THK5117 and ^3^H-deprenyl on frozen whole left hemisphere sections showing selections within the temporal gyrus (A-D for ^3^H-THK5117 and A’-D’ for ^3^H-deprenyl) for AD brains A, B and C and the control brain D. EC: entorhinal cortex, FFG: fusiform gyrus and ITG: inferior temporal gyrus. The regions of interest chosen for the quantitative analysis are highlighted in yellow.


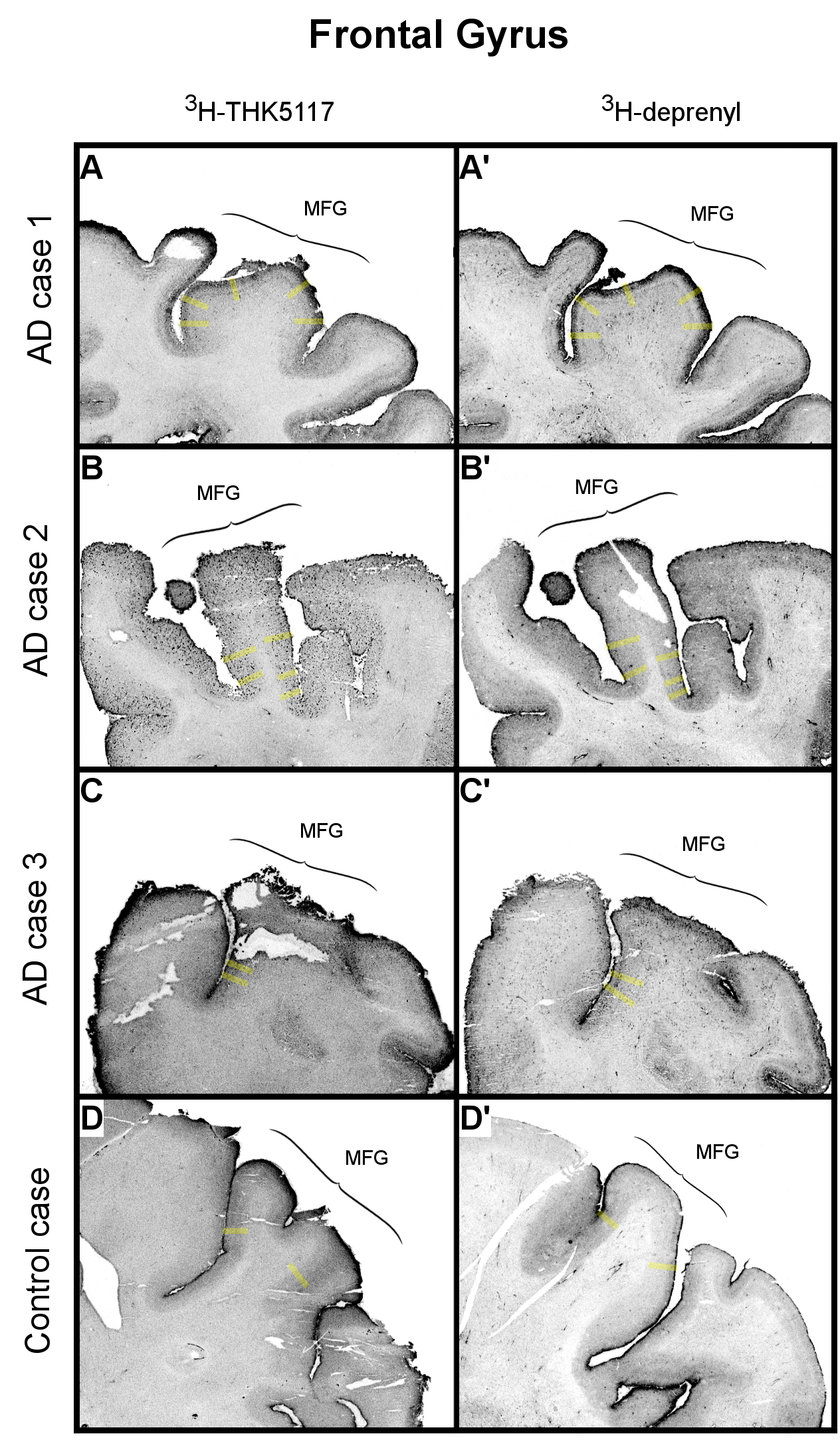


**Supplementary Figure S2.** Autoradiography with ^3^H-THK5117 and ^3^H-deprenyl on frozen whole left hemisphere sections showing selections within the frontal gyrus (A- D for ^3^H-THK5117 and A’-D’ for ^3^H-deprenyl) for AD brains A, B and C and the control brain D. MFG: middle frontal gyrus. The regions of interest chosen for the quantitative analysis are highlighted in yellow.


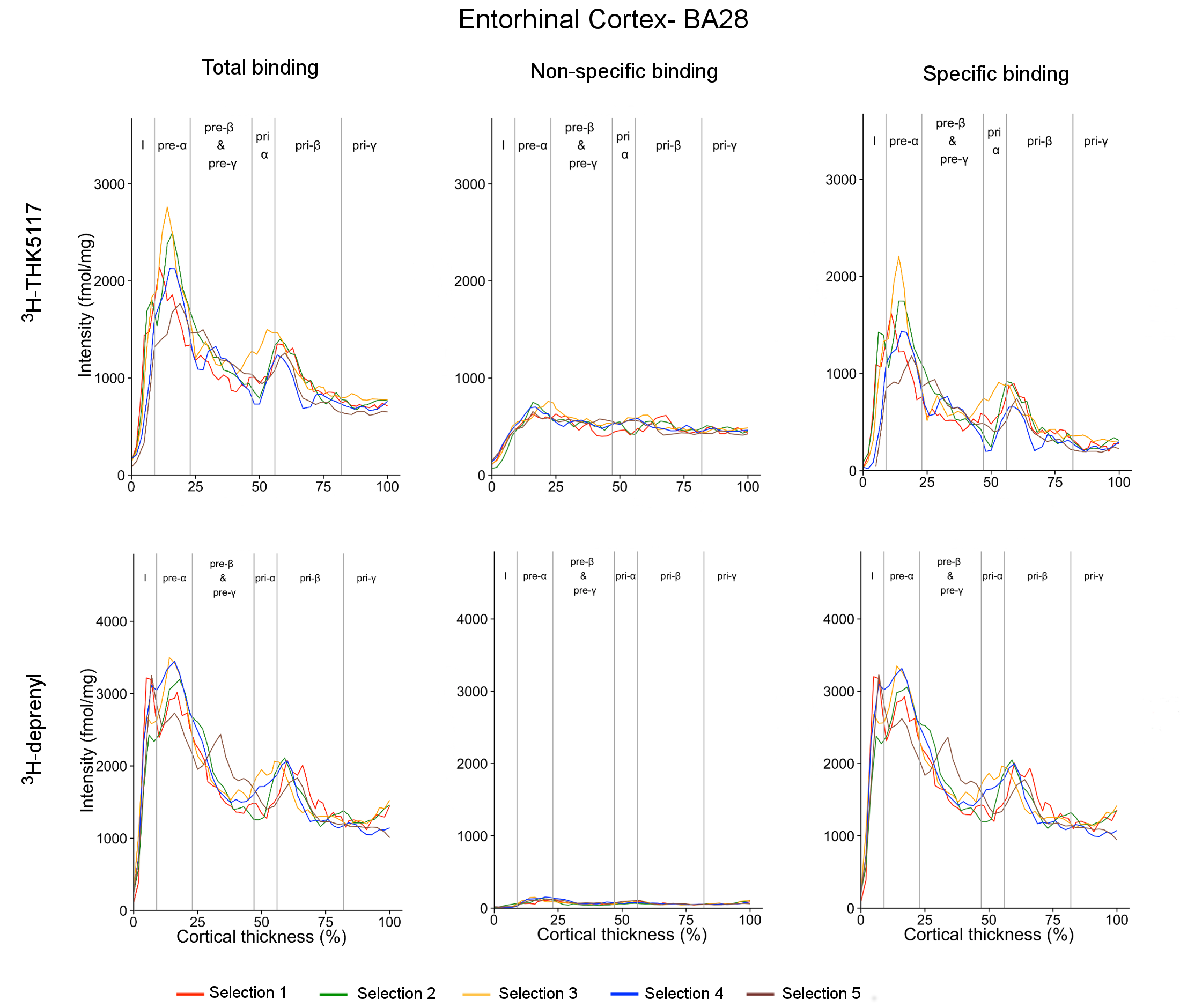


**Supplementary Figure S3.** Laminar binding profiles for total, non-specific and specific binding to the entorhinal cortex in AD brain A with ^3^H-THK5117 in the upper panel and ^3^H-deprenyl in the lower panel. The different colors correspond to different selection in the region of interest.

Supplementary Data 1


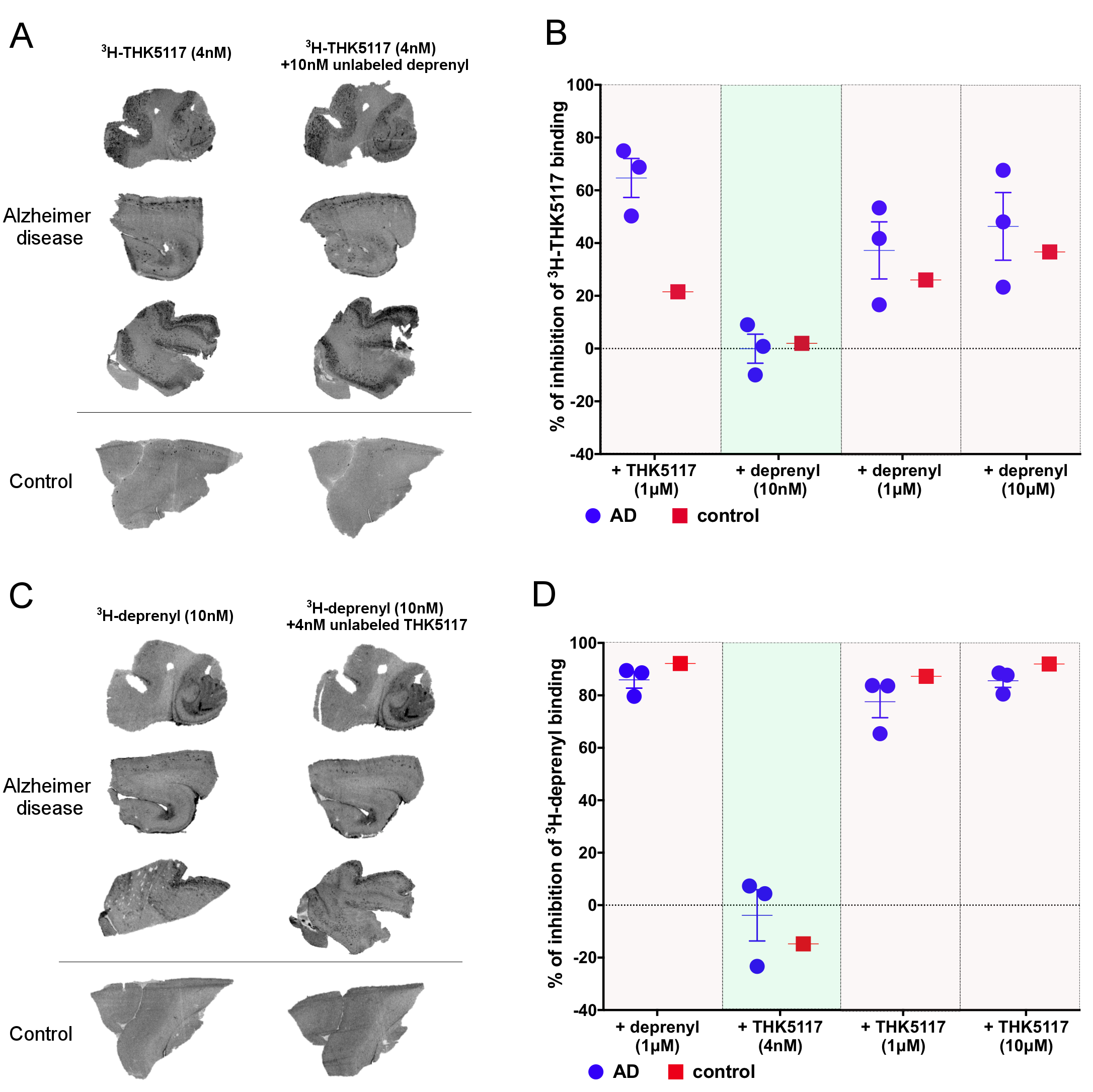


**Figure** **legend:** Competition autoradiography between THK5117 and

Deprenyl in hippocampal region from three AD and one control case. A shows the autoradiographies from each case using ^3^H-THK5117 (4nM) in the left panel, and the competition with unlabeled deprenyl at 10nM in the right panel. B shows the percentage of inhibition using the unlabel compound (THK5117 10μM; deprenyl 10nM (highlighted in green concentration used in the manuscript); deprenyl 1μM; deprenyl 10μM). C shows the autoradiographies from each case using ^3^H-deprenyl (10nM) in the left panel, and the competition with unlabeled THK5117 at 4nM in the right panel. D shows the percentage of inhibition using the unlabel compound (deprenyl 1μM; THK5117 4nM (highlighted in green concentration used in the manuscript); THK5117 1μM; THK5117 10μM)

**Experimental procedures:** Competition autoradiography studies were performed on small frozen sections from three AD cases and one control case in the hippocampal region (see details in the table below), in order to check the selectivity of our tracers towards each other. Similar protocol as described previously was applied. ^3^H-THK5117 (4nM) autoradiography were performed in competition with 10nM, 1μM and 10μM of unlabeled deprenyl. ^3^H-deprenyl (10nM) autoradiography were performed in competition with 4nM, 1μM and 10μM of unlabeled deprenyl.

Demographic table for the supplementary data:

|  | Braak stage | Age | Gender |
| --- | --- | --- | --- |
| AD case | 6C | 70 | M |
| AD case | 6C | 79 | F |
| AD case | 4C | 77 | M |
| Control case | 1B | 77 | F |
